# Klinische Ergebnisse von Chlormethin‐Gel bei Mycosis fungoides in “sensiblen” Bereichen: Eine retrospektive Fallserie

**DOI:** 10.1111/ddg.15797_g

**Published:** 2025-11-14

**Authors:** Gianluca Tavoletti, Gianluca Avallone, Pamela Vezzoli, Paolo Sena, Angelo V. Marzano, Emilio Berti, Silvia Alberti‐Violetti

**Affiliations:** ^1^ Dermatology Unit Fondazione IRCCS Ca' Granda Ospedale Maggiore Policlinico Milan Italy; ^2^ Department of Pathophysiology and Transplantation Università degli Studi di Milano Milan Italy; ^3^ Dermatology Unit ASST Papa Giovanni XXIII Bergamo Italy; ^4^ Inter‐Hospital Pathology Division IRCCS MultiMedica Milan Italy

**Keywords:** Chlormethin, kutane Lymphome, Mycosis fungoides, chlormethine, cutaneous lymphomas, Mycosis fungoides

Sehr geehrte Herausgeber,

Chlormethin (CL)‐Gel ist das erste topische Therapeutikum, das zur Behandlung der Mycosis fungoides (MF) entwickelt wurde.^1^ In internationalen Leitlinien wird es zur Erstlinientherapie für erwachsene Patienten mit MF im Frühstadium (IA‐IIA) und in Kombination mit systemischen Therapien für Patienten in fortgeschrittenen Stadien empfohlen.^2^, ^3^, ^4^ Obwohl klinische Studien und Evidenz aus der Praxis seine Wirksamkeit belegen,^5^, ^6^ ist die Anwendung von CL‐Gel an anatomisch “sensiblen” Stellen (wie Gesicht, Anogenitalbereich und Intertrigines) aufgrund des Risikos lokaler unerwünschter Ereignisse (AEs), insbesondere irritativer Kontaktdermatitis (*irritant contact dermatitis*, ICD) schwierig.^7^ Obwohl einige anekdotische Belege darauf hindeuten, dass CL‐Gel an solchen Stellen verwendet werden kann,^8^, ^9^ besteht ein Bedarf an realen Daten, um seine Wirksamkeit zu belegen und Behandlungsstrategien für damit verbundene AEs zu beschreiben.

Von Januar 2020 bis April 2024 führten wir eine retrospektive Fallserie an zwei italienischen tertiären Referenzzentren durch. Die Studie wurde von der Ethikkommission Milano Area 2 genehmigt (Protokollnummer: 0007202). Die in Frage kommenden Patienten hatten eine histologisch bestätigte MF‐Diagnose mit mindestens einem betroffenen “sensiblen” Bereich und wurden mit CL‐Gel behandelt. Die Untersuchungen erfolgten zu Beginn der Behandlung und alle 3 Monate nach anerkanntem Standard und institutionellen Protokollen. Das Ansprechen wurde anhand eines *modifizierten Composite Assessment of Index Lesion Severity* (mCAILS) bewertet, bei dem die Hyperpigmentierung ausgeschlossen wurde.^7^, ^8^ Die objektive Ansprechrate (*objective response rate*, ORR), definiert als der Anteil der Patienten, die bei der Bewertung nach drei Monaten entweder ein vollständiges (mCAILS = 0) oder ein teilweises Ansprechen (≥ 50% Verringerung des mCAILS) erreicht hatten,^6^ wurde berechnet. Die Nebenwirkungen wurden nach den *Common Terminology Criteria for Adverse Events, Version 5.0, des National Cancer Institute* eingestuft.^10^

Wir schlossen acht Patienten (5 Männer und 3 Frauen) mit MF in verschiedenen Stadien ein (75% im Frühstadium) (Tabelle 1). Die Patienten waren im Median 56,5 Jahre alt (Q1 = 50; Q3 = 58,5), und das mediane Alter bei der Diagnose betrug 43,5 Jahre (Q1 = 40; Q3 = 49). Klinische Varianten waren klassische MF (n = 5), follikulotrope MF (n = 2) und poikilodermatische MF (n = 1). “Sensible” Bereiche waren Augenlider (n = 3), Leiste, Wange, Perineum, Schamgegend, Penis und Scrotum. Vier Patienten (50%) verwendeten gleichzeitig topische Kortikosteroide (TCS), um die lokale Reizung zu verringern, und drei Patienten (38%) erhielten eine systemische Therapie. Zwei Patienten (25%) wendeten das CL‐Gel täglich an und sechs Patienten (75%) applizierten es jeden zweiten Tag. Die mediane Behandlungsdauer betrug 3 Monate (Q1 = 2; Q3 = 3,5). Nach drei Monaten erreichten sechs Patienten (75%) die ORR, während zwei Patienten eine persistierende Erkrankung hatten, deren Schweregrad jedoch abnahm. Bei den Patienten, die sich einer systemischen Therapie unterzogen, wurde das CL‐Gel gezielt bei rezidivierenden oder neuen Läsionen eingesetzt, was die allgemeine Krankheitskontrolle weiter unterstützte. Bei einer medianen Nachbeobachtungszeit von 22 Monaten (Q1 =  14; Q3 = 42) erreichten sechs Patienten ein vollständiges Ansprechen (mCAILS = 0), während zwei (25%) eine minimale Restkrankheit aufwiesen (Abbildung 1). Unerwünschte Ereignisse traten bei sechs Patienten (75%) auf, darunter leichte ICD bei drei Patienten (38%), mäßige ICD bei zwei (25%) und schwere ICD bei einem (13%). Bis auf einen Patienten setzten alle anderen das CL‐Gel nach kurzer Unterbrechung oder reduzierter Anwendungshäufigkeit fort. Zur Behandlung von Entzündungen wurden begleitend TCS eingesetzt, wobei die Ärzte aufgrund des erhöhten Risikos einer steroidbedingten Atrophie in sensiblen Bereichen vorsichtig blieben. Bei sechs Patienten (75%) wurde in den behandelten Bereichen eine vorübergehende Hyperpigmentierung beobachtet.

**TABELLE 1:** Zusammenfassung der Patientenmerkmale.

Patient Nr.	1	2	3	4	5	6	7	8
Alter/Geschlecht	46/M	57/M	42/M	81/M	59/F	54/F	56/F	58/M
Alter bei der MF‐Diagnose (Jahre)	38	42	19	68	46	48	50	43
Fitzpatrick‐Hauttyp	II	III	III	III	III	III	III	II
Klinische Varianten	Klassische MF	Poikilodermatische MF	Klassische MF	Klassische MF	Follikulotrope MF	Klassische MF	Follikulotrope MF	Klassische MF
Frühere MF‐Therapien	TCS, nb‐UVB, Aci	TCS, PUVA, nb‐UVB	TCS, Aci,	TCS, nb‐UVB, RT	TCS, PUVA, Aci, Bexa, Ifn, RT	TCS, PUVA, Aci, RT	TCS, nb‐UVB, Aci, PUVA	TCS, nb‐UVB, Aci
Alter bei CL‐Beginn	44	40	41	80	55	50	54	57
Stadium bei CL‐Beginn	Ia	Ia	Ib	Ib	IIb	Ib	IIb	IIa
Betroffene sensible Bereiche	Leiste	Perineum	Augenlid	Schambereich	Augenlid	Augenlid	Wange	Penis und Scrotum
Art der behandelten Läsion	Ekzemartig	Plaque	Ekzemartig	Plaque	Plaque	Plaque	Plaque	Plaque
Häufigkeit der CL‐Anwendung	Jeden zweiten Tag	Jeden zweiten Tag	Jeden Tag	Jeden zweiten Tag	Jeden zweiten Tag	Jeden Tag	Jeden zweiten Tag	Jeden zweiten Tag
Dauer der CL‐Behandlung	3 Monate	4 Monate	5 Tage	2 Monate	3 Monate	9 Monate	3 Monate	2 Monate
topische Kombinationstherapien	TCS	TCS	Keine	TCS	Keine	Keine	Keine	TCS
systemische Kombinationstherapien	Keine	Keine	Keine	Keine	Bexa	keine	PUVA	Aci
mCAILS bei CL‐Einführung (T0)	16	20	11	22	12	13	15	11
mCAILS bei T3	7	9	0	0	0	8	2	6
mCAILS bei T6	0	0	0	0	0	5	0	3
mCAILS bei T12	0	0	0	0	0	3	0	3
Monate bis zur letzten Nachuntersuchung	36	14	14	24	48	48	24	12
mCAILS bei der letzten Nachuntersuchung	0	0	0	0	0	3	0	3
Hyperpigmentierung	Ja	Ja	Ja	Ja	Ja	Nein	Ja	Nein
Unerwünschte Ereignisse	ICD (leicht)	ICD (mäßig)	ICD (schwer)	ICD (mäßig)	ICD (leicht)	Keine	Keine	ICD (leicht)
Management von unerwünschten Ereignissen	TCS	zeitweises Absetzen	Absetzen, TCS	zeitweises Absetzen, reduzierte Anwendungsfrequenz, TCS	zeitweises Absetzen	Keine	Keine	reduzierte Anwendungsfrequenz

*Abkürzungen*: TCS, topische Kortikosteroide; nb‐UVB, Schmalband‐Ultraviolett B; PUVA, Psoralen und Ultraviolett A; RT, Strahlentherapie; Aci, Acitretin; Bexa, Bexaroten; Ifn, Interferon; mCAILS, modifizierte *Composite Assessment of Index Lesion Severity*; ICD, irritative Kontaktdermatitis; MF, Mycosis fungoides; CL, Chlormethin

**ABBILDUNG 1:**
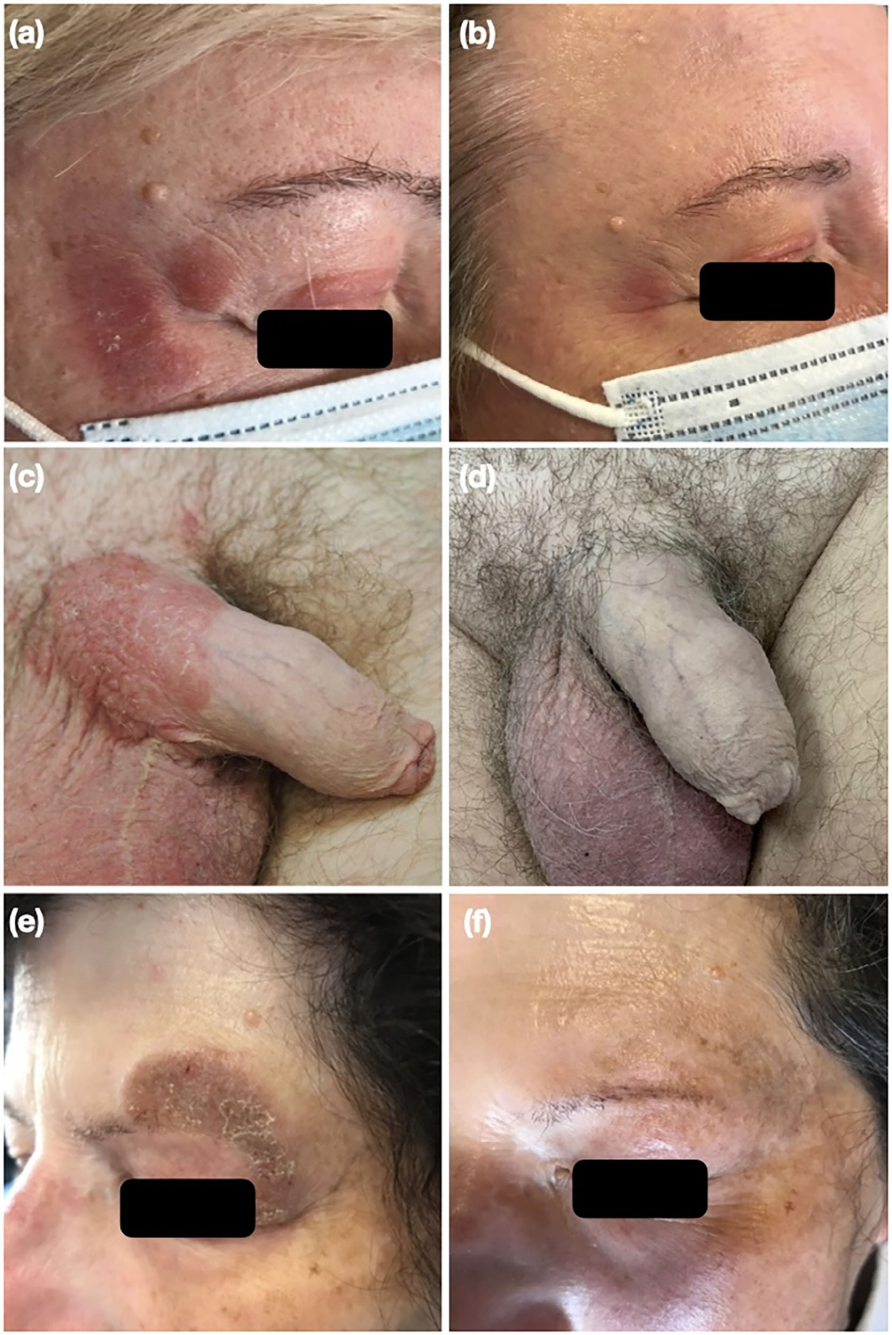
Patienten mit Mycosis fungoides in sensiblen Bereichen, die mit Chlormethin‐Gel behandelt wurden: (a, c, e) Ausgangszustand und (b, d, f) Aussehen bei der letzten Nachuntersuchung.

Obwohl klinische Studien darauf hindeuten, dass in sensiblen Bereichen ein erhöhtes Risiko für Nebenwirkungen bei der Anwendung von CL‐Gel besteht,^7^, ^8^ zeigt diese retrospektive Fallserie, dass CL‐Gel bei MF‐Läsionen in empfindlichen Hautregionen sowohl sicher als auch wirksam sein kann. Die gleichzeitige Anwendung von TCS scheint die Verträglichkeit zu verbessern, was mit früheren Beobachtungen übereinstimmt.^6^

Zu den Einschränkungen der Studie gehören das retrospektive Design, die relativ kleine Stichprobengröße und die ausschließliche Einbeziehung hellhäutiger Patienten. Künftige prospektive Studien sollten die optimalen Anwendungszeitpläne untersuchen, unterstützende Maßnahmen zur Vorbeugung oder Behandlung von ICD bewerten und die Rolle von CL‐Gel in Kombination mit systemischen Wirkstoffen weiter klären.

## DANKSAGUNG

Open access publishing facilitated by Universita degli Studi di Milano, as part of the Wiley ‐ CRUI‐CARE agreement.

## INTERESSENKONFLIKT

Keiner.
